# Epidemiology of fractures in Armenia: development of a country-specific FRAX model and comparison to its surrogate

**DOI:** 10.1007/s11657-017-0392-6

**Published:** 2017-11-07

**Authors:** O. Lesnyak, S. Sahakyan, A. Zakroyeva, J. P. Bilezikian, N. Hutchings, V. Babalyan, R. Galstyan, A. Lebedev, H. Johansson, N. C. Harvey, E. McCloskey, John A. Kanis

**Affiliations:** 1 Mechnikov North West State Medical University, 41, Kirochnaya Street, St. Petersburg, 191015 Russia; 2 Artashat Medical Center, 7, Aram Khachaturyan Street, 0701 Artashat, Armenia; 3 Ural State Medical University, 3 Repina Street, Yekaterinburg, 620028 Russia; 4 College of Physicians and Surgeons, Columbia University, 630 W. 168th Street, New York, NY 10032 USA; 5 “Diavant” LLC, 7 Argishti Street, 0015 Yerevan, Armenia; 6 Scientific Center of Traumatology and Orthopedics of the Ministry of Health of the Republic of Armenia, 9, Nork-Marash Street, 0047 Yerevan, Armenia; 7 Institute for Health and Ageing, Catholic University of Australia, Melbourne, Australia; 8 MRC Lifecourse Epidemiology Unit, University of Southampton, Southampton, UK; 9 Centre for Metabolic Bone Diseases, University of Sheffield, Sheffield, UK

**Keywords:** FRAX, Fracture, Fracture probability, Epidemiology, Hip fracture, Forearm fracture, Humerus fracture

## Abstract

***Summary*:**

Fracture probabilities derived from the surrogate FRAX model for Armenia were compared to those from the model based on regional estimates of the incidence of hip fracture. Disparities between the surrogate and authentic FRAX models indicate the importance of developing country-specific FRAX models. Despite large differences between models, differences in the rank order of fracture probabilities were minimal.

**Objective:**

Armenia has relied on a surrogate FRAX model based on the fracture epidemiology of Romania. This paper describes the epidemiology of fragility fractures in Armenia used to create an Armenia-specific FRAX model with an aim of comparing this new model with the surrogate model.

**Methods:**

We carried out a population-based study in two regions of Armenia (Ararat and Vayots Dzor representing approximately 11% of the country’s population). We aimed to identify all low-energy fractures: retrospectively from hospital registers in 2011–2012 and prospectively in 2013 with the inclusion of primary care sources.

**Results:**

The differences in incidence between the surveys with and without data from primary care suggested that 44% of patients sustaining a hip fracture did not receive specialized medical care. A similar proportion of forearm and humeral fractures did not come to hospital attention (48 and 49%, respectively). Only 57.7% of patients sustaining a hip fracture were hospitalized. In 2013, hip fracture incidence at the age of 50 years or more was 201/100,000 for women and 136/100,000 for men, and age- and sex-specific rates were incorporated into the new “authentic” FRAX model for Armenia. Compared to the surrogate model, the authentic model gave lower 10-year fracture probabilities in men and women aged less than 70 years but substantially higher above this age. Notwithstanding, there were very close correlations in fracture probabilities between the surrogate and authentic models (> 0.99) so that the revisions had little impact on the rank order of risk.

**Conclusion:**

A substantial proportion of major osteoporotic fractures in Armenia do not come to hospital attention. The disparities between surrogate and authentic FRAX models indicate the importance of developing country-specific FRAX models. Despite large differences between models, differences in the rank order of fracture probabilities were minimal.

## Introduction

In 2008, the WHO Collaborating Centre for Metabolic Bone Diseases at the University of Sheffield, UK, developed algorithms to compute age-specific fracture probabilities in women and men from readily obtained clinical risk factors (CRFs) and BMD measurements at the femoral neck (http://www.shef.ac.uk/FRAX). The algorithm (FRAX®) was based on a series of meta-analyses using the primary data from population-based cohorts that identified several CRFs for fracture [1, 2]. FRAX models compute the probability of major osteoporotic fracture (hip, spine, distal forearm, or proximal humerus) or hip fracture derived from the risk of fracture and the competing risk of death, both of which vary from country to country [3]. At present, FRAX models are available for 64 countries.

Ideally, data pertinent to both fracture incidence and death should be available for construction of country-specific FRAX models. Recognizing that data on hip and other fractures are not always available, the International Society for Clinical Densitometry and International Osteoporosis Foundation recommended utilizing a surrogate FRAX model, based on the country-specific risk of death and fracture data based on a country where fracture rates were considered to be representative of the index country [4]. Of the 63 countries for which a FRAX model is available, four FRAX country-specific models use surrogate data on fracture risk (Sri Lanka, India, Palestine, and until recently, Armenia). In the case of Armenia, Romania was used as a surrogate country for its FRAX model.

The aim of this study was to develop an authentic FRAX model for Armenia, because of recently acquired population-based data on fractures and to compare this with the FRAX model based upon the surrogate version.

## Methods

Armenia is a landlocked country located in the Transcaucasian region. It is bordered on the north by Georgia, the east by Azerbaijan, the south by Iran, and the west by Turkey. The total population in 2015 was estimated at 3,018,000. The majority of Armenia’s population (66.8%) is concentrated in the cities of Yerevan (the capital) and Gyumri in the western and northwestern parts of the country, respectively, and other cities. Only 33.2% live in rural areas. The gross domestic product (GDP) per capita in Armenia was last recorded at US$7907 in 2015, when adjusted by purchasing power parity (PPP). WHO statistics indicate that total expenditure on health was 4.4% of GDP in 2007, and average life expectancy from birth was 71 years [5, 6].

The robustness of the epidemiological data for fracture depends in part on identifying all fractures within a defined catchment area. Where every fracture leads to a hospitalization or medical attention, or becomes part of a national health registry, this is a straightforward task. There was reason to assume, however, that not all fractures in Armenia lead to hospitalization or even outpatient medical attention. This phenomenon of “escaping official statistics,” particularly with regard to hip fracture, has been reported in other countries in the region including Russia [7], Georgia, Kazakhstan, and Kyrgyzstan [8]. In the case of Russia, the underreporting of fractures was quantified by means of a survey in which information on hip fracture was obtained not only from orthopedic services but also from primary care physicians [7]. The same approach was used in the “Epidemiology of osteoporotic fractures in Eurasian counties” study (EVA or ЭВА, in Russian) of which the present study is a component.

Two regions of Armenia were identified to document the incidence of fractures attributable to osteoporosis—Ararat region and Vayots Dzor region. The Ararat region covers 2096 km^2^ and is located on the Western Armenian border, near Turkey. Vayots Dzor region, the least populated in Armenia, is in the southeast, covering an area of about 2300 km^2^. It is 123 km from Yerevan. The catchment sizes that formed the basis for calculations of fracture incidence were provided by the National Statistics Service. The population in both regions is predominantly rural and the vast majority is of Armenian ethnicity (96.6%). For the relevant years, the populations of the Ararat and Vyots Dzor regions were 284,574 and 52,252, respectively. Overall, these regions represent about 11% of the entire Armenian population. In both regions, in 2011, the population of 50 years or older was 45,871 for men and 55,838 for women [9]. These regions were chosen because of well-defined catchment populations which were problematic in urban areas of Armenia.

In the first phase of the study, we retrospectively collected information covering a 2-year period, 2011–2012, from official records of all trauma services of the two regions provided by seven hospitals including inpatient and outpatient clinics. The retrospective survey covered a 24-month period (2011–2012) for documentation of humeral (International Classification of Diseases [ICD]-10 code S 42.2), forearm (S52.5, S52.6), and hip (S72.0, S72.1, S72.2) fractures. We evaluated the registries of hospitalizations in hospital admission departments and outpatient orthopedic units in the seven hospitals as well as the records of the regions’ emergency services. Fractures were documented in men and women aged 50 years or older according to ICD codes and were validated from the medical records and radiographs. We reviewed medical records to check level of trauma and place of residence. We included only residents of the Ararat and Vyots Dzor regions. Pathological fractures and high-energy fractures such as following falls from greater than a standing height were also excluded. If the patient developed the same type of fracture again, during this period, it was registered as a new event.

The second phase of the study was prospectively undertaken in 2013. In addition to the methods of fracture acquisition detailed above, we surveyed all community sources and general practices to capture data on fracture patients including those who did not seek hospital-based orthopedic care, and thus, were not registered in the records of the orthopedic service. The records of home visits by orthopedic surgeons were examined. Additionally, letters were addressed to all 71 general practitioners to identify low-mobility or bedridden elderly persons among their patients that might have sustained a hip fracture. Candidates were visited and examined by an orthopedic surgeon and, where possible, underwent X-ray examination. In 16 frail elderly hip fracture patients, X-ray examination was not possible and the presence or absence of hip fracture was based on a clinical diagnosis by the orthopedic surgeon.

We excluded a second admission in the observation period for the same fracture site. All health care institutions providing medical care in each region were included in the study, and data on all the available cases of hip, distal forearm, and proximal humerus in inhabitants of 50 years and older were collected. In all, the fracture rate for 2013 was ascertained from a survey of 7 hospitals and 71 primary care centers.

Age- and sex-specific incidence of hip, forearm, and humeral fractures were calculated in 5-year age intervals from the age of 50 years. We standardized age- and sex-specific rates from the two regions to the entire population of Armenia for 2010 using the medium variant of United Nations population data. Thereafter, the data from the two regions were combined, weighted by population size, to compute the age- and sex-specific incidence of hip, forearm, and humeral fractures.

Data on clinical vertebral fracture were not collected. On the assumption that the age- and sex-specific patterns of fractures due to osteoporosis are similar in different communities [10, 11], as has been done routinely for the majority of FRAX models, we imputed rates from the epidemiology of such fractures in Sweden [12]. This assumes that the age- and sex-specific ratios of clinical spine fracture and hip fracture in Armenia are similar to those seen in Sweden. The adequacy of the ratios was tested, in the case of forearm and humeral fractures, by comparing empirical ratios from Armenia with those derived from Sweden [12] when both were applied to the age-standardized population of Armenia (2010).

### Comparison of models

The Armenian FRAX model incorporated both fracture and death hazards relevant for Armenia (termed the authentic FRAX model) whereas the Romanian surrogate FRAX model used the fracture rates of Romania but with the death risk of Armenia. For the purpose of comparing the authentic FRAX model and the surrogate, the probabilities of a major osteoporotic fracture (hip, clinical spine, forearm, and humeral fractures) and of hip fracture alone were computed in men and women at ages 50, 60, 70, and 80 years for all possible combinations of clinical risk factors at BMD *T* scores between 0 and − 3.5 SD in 0.5 SD steps with a BMI set to 25 kg/m^2^ [13]. Thus, we considered all combinations of six risk factors and eight values of BMD giving a total number of combinations of 512. Note that this was not a population simulation, but an array of all possible combinations. The correlation between the probabilities derived from the surrogate and authentic models was examined by piecewise linear regression with knots at the probabilities of 10 and 30% for the Armenia surrogate probabilities of a major osteoporotic fracture and at 5 and 20% for hip fracture. The reason for using knots at different probabilities for the two outcomes was because of differences in the distribution of probabilities. Tabular data were used to compare probabilities between the two versions at the 10th, 50th (median), and 90th percentile of the distribution of the surrogate model. Differences in the authentic model from the surrogate model at these percentiles were expressed as 95% tolerance intervals (TI).

## Results

The age-adjusted incidence of fractures was similar in the Ararat and Vayots Dzor regions (data not shown). Therefore, we combined the data from these two regions. Overall, in 2011–2012, we identified 396 low-energy fractures, of which 199 were hip fractures, 130 were forearm, and 67 were humerus fractures.

In 2013, with the added information obtained from primary care centers, the number of identified fragility fractures was almost twice as many as in 2011 or 2012 (Table 1). In all 3 years, the majority of fractures were in women; the crude female/male ratio was 1.8, 3.8, and 2.7 for hip, forearm, and humerus fractures, respectively. Assuming that the difference in annual fracture numbers between 2011/2012 and 2013 was accounted for by non-registered fractures, then the proportion of fractures missed in 2011/2012 was 46% (44, 48, and 49% for hip, distal forearm, and humeral fractures, respectively).

**Table 1:** The number of identified fractures by fracture site during the study periods

	2011			2012			2013		
	Men	Women	Both genders	Men	Women	Both genders	Men	Women	Both genders
Hip	35	58	93	32	74	106	63	114	177
Forearm	10	48	58	15	57	72	26	100	126
Humerus	4	32	36	7	24	31	18	48	66
Total	49	138	187	54	155	209	107	262	369

**Fig. 1:**
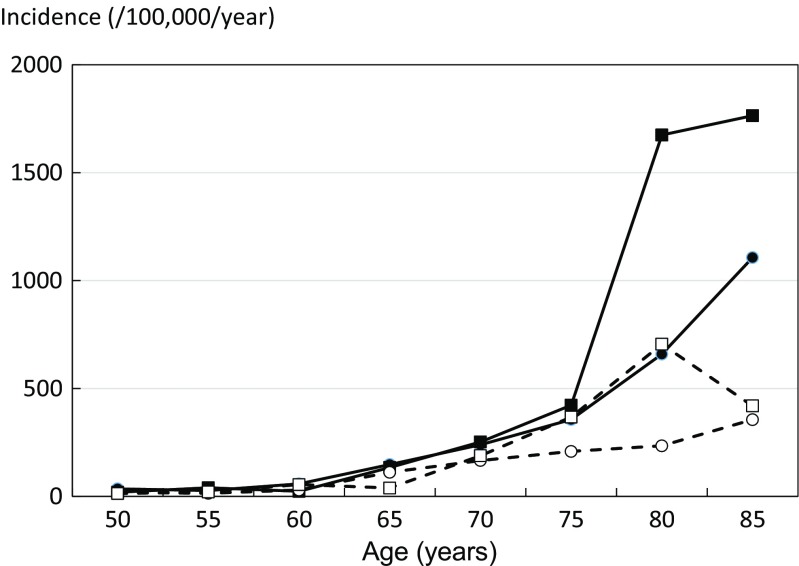
The annual incidence of hip fractures (rate/100,000) by age and sex in Armenia among men (circles) and women (squares). The solid lines and symbols give rates for 2013, and the dashed lines, the rates for 2011 and 2012 combined (square symbols women; circles men)

Hip fracture was the most common type of low-energy fracture. However, among 369 hip fracture patients identified in 2013, only 217 (58.8%) were hospitalized. The increase in the number of identified hip fractures in 2013 was noted in both sexes but was more marked in the elderly (Fig. 1). In 2011–2012, the apparent incidence of hip fractures in those aged 50 years or more was 134/100,000 for women and 73/100,000 for men (female/male ratio 1.8). In 2013, the incidence was 201/100,000 for women and 136/100,000 for men (female/male ratio 1.5). Hip fracture incidence rates increased with age in both sexes, was similar in men and women up to the age of 70 years, and thereafter became much higher in women (Fig. 1). Assuming that fracture rates in the Ararat and Vayots Dzor regions were representative for Armenia, we estimated that the annual number of hip fractures in Armenia was 2067 in 2013.

The forearm was the second most frequent low-energy fracture. Its incidence in 2013 among women and men was 176.4/100,000 and 56.1/100,000, respectively, with a female/male ratio of 3.1. As was the case for hip fracture, the difference between the numbers of detected fractures in 2013 as compared to 2011–2012 suggests that 48.4% of forearm fractures were missed (43.8% for hip fractures). The incidence of humeral fractures (39.2/100,000 for men and 86.0/100,000 for women) and the estimate of missed cases were similar for humerus fracture (49.2%).

The numbers and ratios in the incidence of forearm/hip and humerus/hip fractures in Armenia in 2013 compared to the data of Malmö, Sweden [12], are presented in Table 2. The incidences of both forearm (56.1/100,000 for men and 176.4/100,000 for women) and humeral fractures (39.2/100,000 for men and 86.0/100,000 for women) in 2013 were lower than those in Malmö but the ratios of forearm/hip and humerus/hip were close to those observed in Malmö.

**Table 2:** Annual incidences of hip, forearm, and humeral fracture in men and women (/100,000) from Armenia (2013) and in Malmö, Sweden, [12] age-standardized to the population of Armenia (2010) and the ratios of forearm/hip and humerus/hip

	Armenia		Malmö	
	Men	Women	Men	Women
Hip	136	201	307	799
Forearm	56	176	147	692
Humerus	39	86	126	400
Forearm/hip ratio	0.41	0.88	0.48	0.87
Humerus/hip ratio	0.29	0.43	0.41	0.50

### Comparison of models

**Fig. 2:**
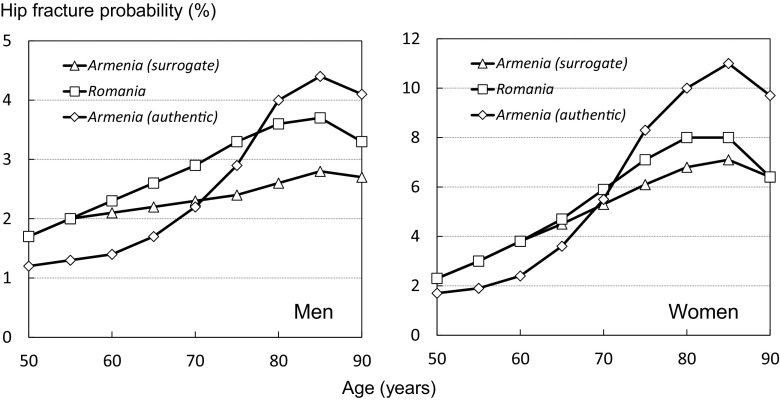
Ten-year hip fracture probability (%) among men (left panel) and women (right panel). Assumes no clinical risk factors, body mass index set at 25 kg/m^2^, no BMD entered

The surrogate Armenian model used hip fracture incidence from Romania. Thus, differences between the surrogate and authentic FRAX models were primarily related to differences in the hip fracture rates between Armenia and Romania. At younger ages, hip fractures in Armenia were lower than those in Romania but increased more steeply with age so that above the age of 70 years, age-specific rates were higher in Armenia than those in Romania. The impact on the 10-year probability of hip fracture is shown in Fig. 2.

### Fracture probability

**Fig. 3:**
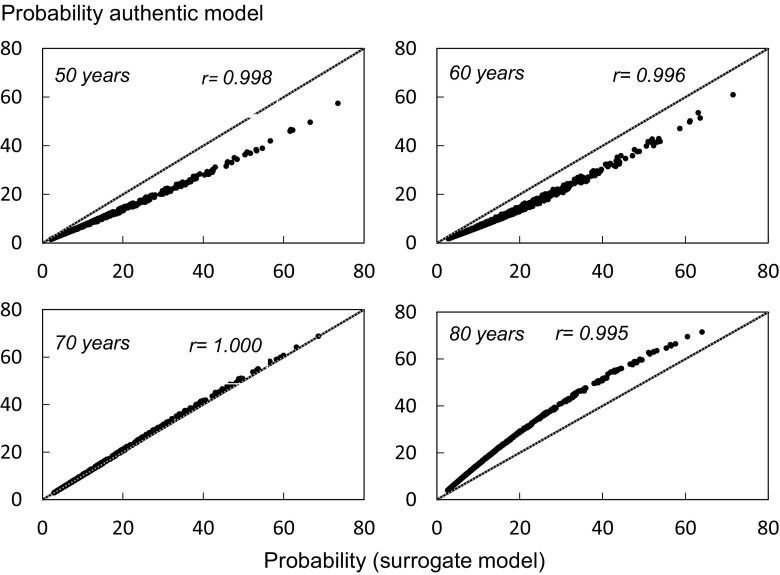
Comparison of 10-year probability of a major osteoporotic fracture using the surrogate FRAX tool for the Armenian female population and the authentic tool for multiple clinical scenarios. The diagonal dashed line shows the line of identity

The relationship between the probabilities of a major fracture derived from the two versions of FRAX is shown for women aged 60 to 80 years in Fig. 3. At all ages, there was a close correlation between the two estimates (*r* > 0.99). The authentic version gave somewhat lower probabilities than the surrogate model at the ages of 50 and 60 years. The median value was lower by 15% at both ages. At the age of 70 years, the slope was very close to the line of identity. At the age of 80 years, the authentic version gave higher probabilities than the surrogate model by 30% at the median value.

In the case of hip fracture, there was also a close correlation between the two estimates (*r* > 0.99) at all ages. The revised version gave lower estimates than the surrogate model at younger ages and higher probabilities at older ages (Table 3). In men, the effect of the revision was qualitatively similar to that in women (Table 4).

**Table 3:** Probability (%) of a major osteoporotic fracture (MOF) or a hip fracture (with 95% tolerance intervals (TI)) in women at the percentiles of the probability distribution (surrogate version) by age

Age	Percentile						*r* value
	10		50		90		
	Surrogate	Authentic (95% TI)	Surrogate	Authentic (95% TI)	Surrogate	Authentic (95% TI)	
MOF							
50	5	4 (3–4)	12	8 (8–9)	31	21 (21–22)	0.998
60	6	4 (3–5)	15	10 (9–11)	35	26 (25–27)	0.996
70	6	6 (6–7)	14	15 (14–15)	35	36 (36–37)	1.000
80	6	9 (9–10)	14	21 (20–21)	36	47 (47–48)	0.995
Hip							
50	0.3	0.2 (0.0–0.4)	2.3	1.4 (1.2–1.6)	20	12.3 (12.0–12.5)	0.999
60	0.5	0.4 (0.3–0.5)	3.1	2.5 (2.4–2.6)	19	15.4 (15.3–15.5)	1.000
70	1.0	1.1 (0.9–1.4)	5.0	5.7 (5.4–5.9)	25	27.3 (27.0–27.6)	1.000
80	1.7	2.7 (1.9–3.6)	7.9	12.2 (11.3–13.1)	30	40.3 (39.4–41.2)	0.995

**Table 4:** Probability (%) of a major osteoporotic fracture (MOF) or a hip fracture (with 95% tolerance intervals (TI)) in men at the percentiles of the probability distribution (surrogate version) by age

Age	Percentile						*r* value
	10		50		90		
	Surrogate	Authentic (95% TI)	Surrogate	Authentic (95% TI)	Surrogate	Authentic (95% TI)	
MOF							
50	4	3 (2–4)	11	8 (7–8)	33	23 (22–23)	0.998
60	4	3 (2–3)	11	8 (7–8)	27	20 (20–21)	0.997
70	3	3 (3–3)	8	8 (8–8)	20	20 (20–20)	1.000
80	3	5 (4–5)	7	11 (10–11)	20	27 (27–28)	0.998
Hip							
50	0.4	0.2 (0.0–0.6)	3.3	2.0 (1.6–2.4)	24	15.2 (14.8–15.5)	0.998
60	0.6	0.5 (0.4–0.5)	3.4	2.7 (2.6–2.7)	17	13.6 (13.5–13.6)	1.000
70	0.9	1.0 (0.7–1.2)	4.0	4.3 (4.0–4.5)	16	16.5 (16.3–16.8)	1.000
80	1.2	1.9 (1.5–2.2)	4.9	7.7 (7.3–8.0)	18	25.4 (25.0–25.7)	0.998

## Discussion

In this study, we documented the incidence of hip, forearm, and humeral fractures in Armenia. The incidence of hip fracture was used to populate an authentic FRAX model to compute the 10-year probabilities of hip and major osteoporotic fractures. The new model can now replace the surrogate model based on hip fracture incidence in Romania. In brief, the revision provided lower estimates of fracture probability at younger ages (50 and 60 years) and higher estimates at older ages (70 and 80 years) than the surrogate model. Importantly, the revisions had little impact on the categorization of risk, since the revisions did not change the rank order of fracture probability. In the clinical scenarios presented in this paper, the correlation coefficients between surrogate and authentic versions for fracture probability exceeded 0.99, so that the one can be accurately predicted from the other. In other words, an individual at the 90th percentile of risk would still be at the 90th percentile of risk using the revised FRAX tool. Thus, the consequences of improving accuracy reside in the absolute number generated and not in the rank order of risk. This is of little consequence to the management of patients or the interpretation of clinical studies. There is a useful analogy with the different DXA devices available, where a substantial difference in femoral neck BMD is seen between Hologic and Lunar machines, but the *T* score derived from these is more or less identical [14]. However, marked difficulties arise when fracture probabilities are used in health economic analysis to inform practice guidelines or devise intervention thresholds.

There are several points of interest with regard to fracture risk. The hospital surveys undertaken in 2011 and 2012 yielded much lower estimates of hip fracture incidence than the survey of 2013, which included primary care contacts. Thus, Armenia joins the several countries (Russia [7], Georgia, Kazakhstan, and Kyrgyzstan [8]) where hip fracture cases do not come to hospital attention. Indeed, 42% of all hip fractures did not receive hospital attention. Of particular interest, the present study suggests that the phenomenon is not confined to hip fracture cases but also pertains to forearm and humeral fractures. The treatment gap arises because patients must pay for hospital admission and/or surgery which they cannot afford. These findings emphasize the importance of exploring treatment pathways in the design of epidemiological studies.

These considerations led us to use the results of the more complete 2013 survey in the synthesis of the Armenian FRAX model. Ideally, FRAX models should use fracture rates for the whole country [15], whereas the present study sampled fracture rates from two regions representing only 11% of the total population. It is well established that there are regional variations in hip fracture rates within countries [12, 16–22], but, given the absence of national registers and “missing cases,” we had to rely on the regional estimates. The situation is not unique and regional estimates have also been used to create FRAX models for Brazil [23], Croatia [24], Greece [25], Spain [26], Russia [7], and Poland [27, 28].

In the present study, we did not collect data on clinical vertebral fracture. For this reason, the FRAX model relied on hip fracture rates to estimate the incidence of a major osteoporotic fracture. For this purpose, it is assumed that the ratio of hip fracture incidence to other FRAX outcomes (clinical spine, distal forearm, and proximal humerus) is the same in the index country as that documented in Sweden. The ratios for Sweden were derived using national hip fracture data for Sweden and data from Malmö for the other fracture outcomes [12]. Despite a large number of studies that have examined the incidence of fractures by age and sex, there are problems in defining the pattern of fractures in different countries [12]. The available evidence indicates that the incidence of major fractures can be reasonably predicted from the incidence of hip fractures [11, 12, 29]. The present paper provided an opportunity to test this in part since data on humeral and forearm fractures were acquired permitting a comparison of forearm/hip and humerus/hip ratios derived from Armenia and Sweden. Within the limitations of the analysis (requires information on the first humeral, forearm, and hip fracture), the pattern of osteoporotic fractures appears to be broadly similar in Armenia and Sweden.

A comparison of probabilities of the authentic model with the surrogate model is given in Table 5 together with neighboring countries where a FRAX model is available. The probabilities were lower than in neighboring countries with Poland being the closest.

**Table 5:** Ten-year probability of major osteoporotic fracture (MOF) and hip fracture in men and women aged 65 years with a prior fragility fracture (body mass index set to 25 g/m^2^; no BMD entered)

Country	Men		Women	
	MOF	Hip fracture	MOF	Hip fracture
Armenia	3.5	1.1	7.3	2.3
Armenia (surrogate)	4.5	1.2	9.1	2.5
Romania	5.2	1.5	9.5	2.6
Russia	9.2	1.3	18	2.6
Poland	4.5	1.2	8.3	2.2
Iran	6.4	2.1	11	3.7
Turkey	5.8	1.4	10	2.3

The importance of creating and calibrating the original FRAX model to an individual country is illustrated by the marked differences in 10-year risk of hip fracture in 50-year-old males and females when the surrogate country for Armenia, namely Romania, is compared to these newly acquired data from Armenia [30]. We found that the surrogate FRAX model slightly overestimates observed fracture risk for people less than 70 years but considerably underestimates (by twofold) the actual hip fracture risk for men and women greater than 70 years. The results of this study should encourage other countries that are employing surrogate countries for their FRAX model to obtain their own country-specific data. Country-specific data are likely to be more accurate and different from the surrogate model. In general, our results indicate that osteoporotic fracture is a common and important threat to the health and independence of the Armenian people in Armenia. The probability of hip fracture is twice as great in women, but it is also common in Armenian men.

Whereas the fracture rates we documented are relatively robust, the extrapolation of these rates to the entire country could be problematic. In addition to large variations in fracture rates around the world, fracture rates may vary within countries with the differences in lifestyle between ethnic groups as well as between urban and rural areas [18]. The potential association of education and marital status with hip fracture incidence in older individuals is of note also [31]. In addition to differences between the countryside and the city [26, 32, 33], differences in hip fracture incidence have been reported using common methodology with the higher rates in urban communities in Argentina [19], Sweden [12], Norway [20] Switzerland [21, 22], Croatia [24], the USA [17], and China [18]. Thus, the fracture probabilities and the fracture projections we report are based on the assumption that the Ararat and Vyots Dzor regions are the representative populations for the entire country. Based on available surveys elsewhere, it is possible that the present regional study might underestimate fracture incidence in urban settings, particularly since approximately 65% of Armenia’s population is concentrated in the cities [6, 9].

Despite the rigor of the methodology, it is possible that not all fractures were captured in the prospective study that formed the database. On the other hand, accuracy errors have little impact on the rank order with which the FRAX tool categorizes risk in a given population [10, 15], but they do change the absolute number generated and thus have implications where treatment guidelines are based on cost-effectiveness or the economic burden of disease. It is also important to recognize that the FRAX model was generated within the country of Armenia. The extent to which it could apply to diasporic Armenians living throughout the world is uncertain [34].

In summary, a country-specific FRAX model has been developed for the country of Armenia. It is based on a representative population of women and men with prospectively obtained epidemiological data. This model should enhance accuracy of determining fracture probability among the Armenian population in Armenia and help guide decisions about treatment.
